# Persistent Lipophilic Environmental Chemicals and Endometriosis: The ENDO Study

**DOI:** 10.1289/ehp.1104432

**Published:** 2012-03-14

**Authors:** Germaine M. Buck Louis, Zhen Chen, C. Matthew Peterson, Mary L. Hediger, Mary S. Croughan, Rajeshwari Sundaram, Joseph B. Stanford, Michael W. Varner, Victor Y. Fujimoto, Linda C. Giudice, Ann Trumble, Patrick J. Parsons, Kurunthachalam Kannan

**Affiliations:** 1 Division of Epidemiology, Statistics and Prevention Research, *Eunice Kennedy Shriver* National Institute of Child Health and Human Health, National Institutes of Health, Department of Health and Human Services, Rockville, Maryland, USA; 2 Division of Reproductive Endocrinology and Infertility, Department of Obstetrics and Gynecology, University of Utah, Salt Lake City, Utah, USA; 3 Department of Obstetrics, Gynecology and Reproductive Sciences, University of California–San Francisco, San Francisco, California, USA; 4 Department of Family and Preventive Medicine, and; 5 Division of Maternal Fetal Medicine, Department of Obstetrics and Gynecology, University of Utah, Salt Lake City, Utah, USA; 6 Division of Environmental Health Sciences, Wadsworth Center, New York State Department of Health, and the Department of Environmental Health Sciences, University at Albany, State University of New York, Albany, New York, USA

**Keywords:** endocrine-disrupting chemicals, organochlorine pesticides, persistent organochlorine pollutants, polybrominated diphenyl ethers, polychlorinated biphenyls

## Abstract

Background: An equivocal literature exists regarding the relation between persistent organochlorine pollutants (POPs) and endometriosis in women, with differences attributed to methodologies.

Objectives: We assessed the association between POPs and the odds of an endometriosis diagnosis and the consistency of findings by biological medium and study cohort.

Methods: Using a matched cohort design, we assembled an operative cohort of women 18–44 years of age undergoing laparoscopy or laparotomy at 14 participating clinical centers from 2007 to 2009 and a population-based cohort matched on age and residence within a 50-mile catchment area of the clinical centers. Endometriosis was defined as visualized disease in the operative cohort and as diagnosed by magnetic resonance imaging in the population cohort. Logistic regression analysis was used to estimate odds ratios (ORs) and 95% confidence intervals (CIs) for each POP in relation to an endometriosis diagnosis, with separate models run for each medium (omental fat in the operative cohort, serum in both cohorts) and cohort. Adjusted models included age, body mass index, breast-feeding conditional on parity, cotinine, and lipids.

Results: Concentrations were higher in omental fat than in serum for all POPs. In the operative cohort, γ-hexachlorocyclohexane (γ-HCH) was the only POP with a significant positive association with endometriosis [per 1-SD increase in log-transformed γ-HCH: adjusted OR (AOR) = 1.27; 95% CI: 1.01, 1.59]; β-HCH was the only significant predictor in the population cohort (per 1-SD increase in log-transformed β-HCH: AOR = 1.72; 95% CI: 1.09, 2.72).

Conclusions: Using a matched cohort design, we found that cohort-specific and biological-medium–specific POPs were associated with endometriosis, underscoring the importance of methodological considerations when interpreting findings.

Endometriosis is a prevalent gynecologic disorder that is characterized by the presence and growth of endometrial tissue in ectopic sites (Giudice 2010). An equivocal body of evidence has emerged regarding the relation between lipophilic persistent organochlorine pollutants (POPs) and endometriosis, following an initial report in primates (Rier et al. 1993), with subsequent experimental evidence (Birnbaum and Cummings 2002), including exposures during sensitive windows of development (Crain et al. 2008). Three of seven studies that focused on dioxins or dioxin-like compounds and endometriosis have observed significantly higher concentrations in women with endometriosis than in those without the condition (Heilier et al. 2005; Mayani et al. 1997; Simsa et al. 2010). In addition, four of nine studies that focused on polychlorinated biphenyls (PCBs) observed similar findings (Gerhard and Runnebaum 1992; Louis et al. 2005; Porpora et al. 2006, 2009). To our knowledge, endometriosis has been significantly associated with only one class of organochlorine pesticides (OCPs): aromatic fungicides (Cooney et al. 2010).

The weighing of available human data is challenging, largely because of the nuances associated with the clinical diagnosis of endometriosis and methodologic practices that affect interpretation. The clinical gold standard remains disease visualized via laparoscopy or by laparotomy [American Society for Reproductive Medicine (ASRM) 2006; Kennedy et al. 2005]. Currently, there is no established noninvasive biomarker for diagnosis (May et al. 2010). Visualization of disease necessitates clinical sampling strategies that may exclude symptomatic women who do not seek care or undergo surgery, which precludes our understanding of endometriosis at the population level. Other widely recognized methodologic practices that affect research findings include convenience-based sampling, self-reported (yes/no) disease, varying modeling practices for parity or breast-feeding history that may reduce internal doses of lipophilic POPs (LaKind et al. 2009), and reliance on serum or plasma in lieu of quantification in fat, the presumed gold standard for lipophilic chemicals (Johnson-Restrepo et al. 2005; Whitcomb et al. 2005). We designed the Endometriosis: Natural History, Diagnosis and Outcomes (ENDO) Study to further delineate the relation between lipophilic POPs and endometriosis.

## Materials and Methods

*Study design and populations.* A matched cohort design was used to estimate endometriosis in two cohorts. The operative cohort comprised menstruating women 18–44 years of age scheduled for a laparoscopy or laparotomy irrespective of indication at one of 14 participating hospital surgical centers located in the Salt Lake City, Utah, or San Francisco, California, areas between 2007 and 2009. Women were eligible to participate if they had no history of surgically visualized endometriosis (to reduce the likelihood of prevalent disease), no injectable hormone treatment within the past 2 years, not breast-feeding for ≥ 6 months, and no history of cancer. The operative cohort was matched to a population cohort on age and residence within a 50-mile geographic catchment area of the participating surgical centers. The sampling framework for the California site was sampled from the white pages of the telephone directory for the targeted geographic area by Genesys Sampling Systems (Horsham, PA), and the Utah site relied on the Utah Population Database (Huntsman Cancer Institute 2012), which represents 94% of state residents. Women were eligible if they were currently menstruating and did not have a history of visualized endometriosis. The population recruitment strategy located women “at risk” for endometriosis (menstruating) and its diagnosis at one of the participating hospitals for the operative cohort (residence). In both cohorts, a letter of introduction preceded telephone screening and eventual recruitment. Statistical power was determined *a priori* as requiring 450 women in the operative cohort based on a reported endometriosis prevalence of 38% and a 20% relative difference in mean serum PCBs concentrations by endometriosis status (Louis et al. 2005) at the time the study was designed. Given the absence of previous population cohort studies, its size was based upon published studies that reported differences in POPs by endometriosis status.

*Data collection.* In-person baseline interviews were conducted with women, followed by anthropometric assessment using standardized portable stadiometers and electronic scales (Lohman et al. 1988) approximately 2 months before surgery, or 2 months before magnetic resonance imaging (MRI) for the population cohort. For women in the operative cohort, surgeons completed standardized data collection instruments on operative findings, diagnoses, and staging of endometriosis using the revised American Society for Reproductive Medicine classification (ASRM 1997). An algorithm was used to automatically calculate endometriosis severity ranging from minimal to severe (stage 1–4) to avoid bidirectional (over- and understaging severity) errors associated with clinical reporting (Buchweitz et al. 2005; Buck Louis et al. 2011; Weijenborg et al. 2007).

In both cohorts, nonfasting blood (~ 24 mL) and urine (~ 120 mL) specimens were obtained for all women using collection kits determined to be free of POPs. For logistical reasons, we did not require fasting blood specimens. Blank containers were periodically sent to the analytical laboratory to check for contamination; none was found. Depending upon availability and clinical judgment about patient safety, 1–5 g omental fat was obtained from women in the operative cohort by surgeons. At the Utah site, Harmonic® ACE 36P shears and scalpel blades (donated by Ethicon Endo-Surgery, LLC, Cincinnati, OH) were used; primarily, bipolar electrocautery and scissors were used at the California site. Fat specimens were placed into Wheaton brown glass bottles that were cleaned with acetone and hexane before use. Epiploica appendiceal fat was obtained in lieu of omental fat for four women, two from each study site.

Institutional review board approval was obtained from all participating study sites. The women provided full consent before any data were collected, and all were remunerated for their time and travel. A more complete description of the study is provided elsewhere (Buck Louis et al. 2011).

*Operational definitions.* Endometriosis is defined in the operative cohort using the gold standard of visualization (ASRM 2006; Kennedy et al. 2005) and further qualified by histologic confirmation (endometrial glands or stroma and/or hemosiderin-laden macrophages). In the population cohort, endometriosis diagnosed by MRI was mainly ovarian endometriomas.

Definitions for relevant covariates were as follows. Body mass index (BMI) was estimated by dividing measured weight in kilograms by height in meters squared and categorized as underweight (< 18.5), normal (18.5–24.9), overweight (25.0–29.9), obese class I (30.0–34.9), and obese class II+ (≥ 35.0) (National Heart, Lung, and Blood Institute 1998). Income was estimated using Department of Health and Human Services Poverty Guidelines (2007) for the 48 contiguous states and the District of Columbia. Breast-feeding history was derived as a conditional variable based upon parity (nulliparous/parous) and categorized as no prior birth, prior birth but no breast-feeding, and prior birth with breast-feeding.

*Toxicologic analysis.* One laboratory processed and quantified all compounds using gas chromatography (GC)/mass spectrometry (MS) with GC/electron capture detector and GC/high-resolution MS (HRMS) (Johnson-Restrepo et al. 2005, 2007; Sjödin et al. 2004). Serum and fat samples were analyzed for three chemical classes of lipophilic persistent pollutants: *a*) OCPs [hexachlorobenzene (HCB), hexachlorocyclohexane (HCH) and its isomers γ-HCH and β-HCH, oxychlordane, *cis*- and *trans*-nonachlor, *cis*- and *trans*-chlordane, and *p*,*p*´-dichlorodiphenyltrichloroethane (*p*,*p*´-DDT) and its metabolites *p*,*p*´-dichlorodiphenyldichloroethylene (*p*,*p*´-DDE) and *o*,*p*´-DDT]; *b*) polybrominated diphenyl ether (PBDE) congeners 47, 99, 100, 153, 154, and 209; and *c*) PCB congeners 18, 28, 44, 49, 52, 66, 74, 87, 99, 101, 114, 118, 128, 138, 146, 149, 151, 153, 156, 157, 167, 170, 172, 177, 178, 180, 183, 187, 189, 194, 195, 196, 201, 206, and 209. Briefly, serum samples were fortified with isotopically labeled internal standards along with the addition of formic acid (80%) and water for denaturation and dilution of samples using a Gilson 215 liquid hander (Gilson Inc., Middleton, WI).

The samples were extracted by solid-phase extraction (SPE) using a Rapid Trace (Caliper Life Science, Hopkinton, MA) modular SPE system. Removal of coextracted lipids was performed on a silica: silica/sulfuric acid column using Rapid Trace equipment for automation. Final analytical determination of the target analytes was performed by GC/isotope-dilution HRMS employing a Thermo Finnigan MAT95XP (Thermo Fisher Scientific, Bremen, Germany). External calibration standards were analyzed with every set of samples, and recoveries of internal standards were checked against external calibration standards. Three blanks were included in every batch comprising 30 samples. Fat samples were extracted by the Soxhlet extraction procedure, and further details of the methods are given elsewhere (Johnson-Restrepo et al. 2005, 2007).

All concentrations are reported in nanograms per gram of fat or serum after subtracting background. All machine-observed concentrations were used without any substitution of concentrations below the limits of detection (LODs) to avoid introducing biases (Guo et al. 2010; Richardson and Ciampi 2003; Schisterman et al. 2006). Serum lipids were analyzed with enzymatic methods (Akins et al. 1989). Total serum lipids (TL) were estimated as

TL = (2.27 × TC) + TG + 62.3 mg/dL,

where TC denotes total cholesterol and TG denotes triglycerides, and were reported in milligrams per deciliter (Philips et al. 1989). Serum cotinine was quantified using high-performance liquid chromatography/tandem MS using an isotope dilution method and external standard calibration plots (Bernert et al. 2009). Serum cotinine was further categorized as noted above to help identify passive and active exposure using established cut-points (Wall et al. 1988).

*Statistical analysis.* The distributions of all chemicals were inspected and summarized by geometric means and 95% confidence intervals (CIs) and percentiles (median, 75th, 95th). Comparisons of select variables by cohort and endometriosis status were made to assess *a priori* defined select variables (i.e., age, BMI, serum cotinine, and lipids) for the analytic phase. Statistical significance (*p* < 0.05) was determined using the chi-square statistic for categorical data and Student’s *t*-test or Wilcoxon nonparametric test for continuous data.

Logistic regression was used to estimate the unadjusted odds ratio (OR) and corresponding 95% CI for each chemical by biological medium and cohort. CIs that excluded 1 were considered significant. All chemical concentrations were first log (*X* + 1)-transformed to achieve normality and then rescaled by their standard deviations so that ORs could be interpreted per 1-SD change in the log-transformed chemical concentration. All analyses used wet-weight concentrations. Adjusted models included age (in years), breast-feeding history (conditional categorical), BMI (continuous), and cotinine (continuous) (Hediger et al. 2005; Nelson et al. 2006; Sasamoto et al. 2006; Zeyneloglu et al. 1997). Serum lipids (milligrams per deciliter) were also entered into serum models to minimize potential biases associated with automatic lipid adjustment (Schisterman et al. 2005). We also adjusted for breast-feeding conditional on parity, given its uncertain relation with endometriosis. In addition, we conducted a number of sensitivity analyses to assess the consistency of findings by removing parity and breast-feeding from models, varying the diagnostic criteria to require both histologic and visualized disease, restricting diagnosis to endometriosis stages 3–4, and restricting the comparison group to women with a postoperative diagnosis of a normal pelvis in the operative cohort. All analyses were conducted using SAS software (version 9.2; SAS Institute Inc., Cary, NC).

## Results

The operative cohort comprised 495 women scheduled for surgery, and the population cohort comprised 131 women, representing 77% and 79% of eligible women in the target populations, respectively. Twenty-six women had no diagnostic information stemming from the cancellation of 22 (4%) surgeries or 4 (4%) unreadable MRIs in the operative and population cohorts, respectively, and were excluded from all analyses. Despite their different sampling frameworks, few differences were observed between cohorts (data not shown), with the exception of a higher percentage of married women in the operative than in the population cohort (76% vs. 60%, respectively), as previously reported (Buck Louis et al. 2011). The incidence of surgically visualized endometriosis was 41% in the operative cohort, whereas MRI-visualized endometriosis was 11% in the population cohort. Most of the cases in the operative cohort were not severe: 71% had stage 1 or 2, and 29% had stage 3 or 4. However, differences were observed by disease status. In the operative cohort, women with endometriosis were significantly younger, of lower parity, and leaner than women without endometriosis (Table 1). In the population cohort, women with endometriosis were comparable to women without disease, with one important difference—the absence of smokers among women with endometriosis (Table 1).

**Table 1:** Characteristics and endometriosis status of study cohorts [n (%)], ENDO Study (n = 600].

Operative (n = 473)			Population (n = 127)		
Characteristic	Endometriosis (n = 190)	None (n = 283)	Endometriosis (n = 14)	None (n = 113)
Age (years)								
< 30		75 (40)		88 (31)		5 (36)		47 (41)
30–39		83 (44)		120 (43)		5 (36)		40 (35)
≥ 30		32 (17)		74 (26)		4 (29)		26 (23)
Mean ± SD		32.0 ± 6.8*		33.6 ± 7.1		33.1 ± 8.3		32.1 ± 7.8
Parity (no. of live births)								
Never been pregnant		81 (43)		74 (26)		5 (36)		46 (41)
0		22 (12)		25 (9)		1 (7)		10 (9)
1		21 (11)		40 (14)		1 (7)		11 (10)
≥ 2		66 (35)		142 (51)		7 (50)		46 (41)
Mean ± SDa		1.8 ± 1.3*		2.2 ± 1.4		2.6 ± 1.6		2.2 ± 1.5
Breast-feeding conditional on parity								
No, nulliparous		103 (55)		101 (36)		6 (43)		56 (50)
No, parous		20 (11)		37 (13)		1 (7)		9 (8)
Yes, parous		65 (34)		144 (51)		7 (50)		48 (42)
Mean ± SDb		5.0 ± 5.5		5.3 ± 4.9		8.3 ± 2.2		7.5 ± 5.6
BMI (kg/m2)								
< 18.5		8 (4)		5 (2)		1 (7)		7 (6)
18.5–24.9		97 (51)		93 (33)		6 (43)		45 (40)
25.0–29.9		39 (21)		70 (25)		4 (29)		29 (26)
30.0–34.9		17 (9)		56 (20)		1 (7)		16 (14)
≥ 35		28 (15)		55 (20)		2 (14)		16 (14)
Mean ± SD		26.3 ± 7.2**		29.2 ± 8.4		27.4 ± 9.0		27.0 ± 6.7
Cotinine (ng/mL)c								
No exposure (0–9.99)		168 (89)		230 (82)		14 (100)		97 (89)
Passive smoking (10–99.99)		8 (4)		16 (6)		0 (0)		5 (5)
Active smoking (100–299.99)		11 (6)		30 (11)		0 (0)		7 (6)
Heavy smoking (300–595.31)		2 (1)		4 (1)		—		—
Mean ± SD for smokers		152.5 ± 105.8		168.3 ± 110.3		—		113.2 ± 101.3
A total of 22 women in the operative cohort were excluded because their surgeries were canceled, and 4 women in the population cohort were excluded because their MRIs were unreadable. None of the differences in the population cohort achieved significance. aRestricted to 394 gravid women. bRestricted to 264 parous women who breast-fed. cCategorization of cotinine by active and passive smoking status based on criteria of Wall et al. (1988). *p < 0.05, and **p < 0.01, comparing endometriosis status within cohort.								

Table 2 presents the distributions of POPs by biological medium and disease status for each of the cohorts. Two differences emerged: *a*) the upper bound of all tertiles based on wet-weight concentrations was higher for the sum of OCPs (ΣOCPs), ΣPBDEs, and ΣPCBs when measured in fat than when measured in serum, and *b*) the geometric mean serum PBDE and PCB concentrations were slightly higher in women without versus women with endometriosis in the operative cohort, whereas the opposite pattern was observed for the population cohort. Significant mean differences in ΣPBDEs measured in fat were observed for the operative cohort, with higher concentrations for women without endometriosis than for women with endometriosis. The reverse pattern was observed for the population cohort for mean ΣPBDEs in serum. No other patterns were evident for the remaining POPs by endometriosis status. The distributions for all individual chemicals by biological medium, cohort, and endometriosis status are provided in Supplemental Material, Tables 1 and 2 (http://dx.doi.org/10.1289/ehp.1104432). Lipid adjusted fat and serum concentrations also are provided in Supplemental Material, Table 3.

**Table 2:** Chemical distributions (wet weight) by cohorts and endometriosis status [n (%)], ENDO Study (n = 600).

Operative (n = 473)			Population (n = 127)		
Biological medium and chemical grouping (tertiles)	Endometriosis (n = 190)	None (n = 283)	Endometriosis (n = 14)	None (n = 113)
Omental fat (n = 340)								
ΣOCPs (ng/g fat)								
1st, 0.17–7.20		50 (34)		62 (33)		—		—
2nd, 7.22–13.74		50 (34)		62 (33)		—		—
3rd, 13.86–103.85		46 (32)		67 (35)		—		—
Geometric mean (95% CI)		9.11 (7.72, 10.74)		9.69 (8.55, 10.97)		—		—
ΣPBDEs (ng/g fat)								
1st, 2.11–26.41		62 (42)**		51 (26)		—		—
2nd, 26.43–70.69		51 (35)		62 (32)		—		—
3rd, 70.82–3295.72		34 (23)		80 (42)		—		—
Geometric mean (95% CI)		36.41 (30.91, 42.89)**		52.73 (44.95, 61.86)		—		—
ΣPCBs (ng/g fat)								
1st, 3.80–22.25		40 (27)*		73 (38)		—		—
2nd, 22.41–43.19		60 (41)		53 (27)		—		—
3rd, 43.52–1855.19		46 (32)		68 (35)		—		—
Geometric mean (95% CI)		31.71 (27.59, 36.45)		31.10 (27.46, 35.21)		—		—
Serum (n = 599)								
ΣOCPs (ng/g serum)								
1st, 0.00–0.01		63 (33)		95 (34)		3 (21)		34 (30)
2nd, 0.01–0.02		66 (35)		88 (31)		7 (50)		43 (38)
3rd, 0.02–0.81		60 (32)		100 (35)		4 (29)		36 (32)
Geometric mean (95% CI)a		0.01 (0.01, 0.01)		0.01 (0.01, 0.01)		0.01 (0.00, 0.03)		0.01 (0.01, 0.01)
ΣPBDEs (ng/g serum)								
1st, 0.00–0.10		74 (39)		89 (31)		2 (14)*		34 (30)
2nd, 0.11–0.18		61 (32)		93 (33)		5 (36)		41 (36)
3rd, 0.19–4.95		54 (29)		101 (36)		7 (50)		38 (34)
Geometric mean (95% CI)		0.13 (0.11, 0.15)		0.14 (0.13, 0.16)		0.28 (0.16, 0.51)*		0.15 (0.13, 0.18)
ΣPCBs (ng/g serum)								
1st, 0.00–0.15		69 (37)		93 (33)		5 (36)		32 (28)
2nd, 0.16–0.57		63 (33)		90 (32)		2 (14)		45 (40)
3rd, 0.58–391.78		57 (30)		100 (35)		7 (50)		36 (32)
Geometric mean (95% CI)		0.26 (0.20, 0.33)		0.30 (0.25, 0.37)		0.32 (0.15, 0.69)		0.31 (0.24, 0.40)
ΣSerum lipids (mg/dL)								
1st, 316.63–548.9		62 (33)		91 (33)		7 (54)		35 (33)
2nd, 549.28–653.68		64 (34)		95 (34)		3 (23)		33 (31)
3rd, 654.09–1631.52		61 (33)		93 (33)		3 (23)		39 (36)
Geometric mean (95% CI)		593.9 (577.3, 610.9)		609.7 (594.8, 624.9)		575.3 (504.7, 655.9)		609.0 (583.7, 635.4)
—, not applicable (no fat available for population cohort). A total of 22 women in the operative cohort were excluded because their surgeries were canceled, and 4 women in the population cohort were excluded because their MRIs were unreadable. Analyte concentrations rounded to two decimal places. aCorresponding four-decimal-point values are 0.0090 (0.0072, 0.0112), 0.0097 (0.0081, 0.0116), 0.0114 (0.0045, 0.0290), and 0.0100 (0.0077, 0.0132), respectively. *p < 0.05, and **p < 0.01, comparing women by endometriosis status within each cohort.								

**Table 3:** Summary of persistent lipophilic chemicals significantly associated with the odds of an endometriosis diagnosis by biological media and cohorts, ENDO Study (n = 600).

OR (95% CI)			AOR (95% CI)		
Biological medium and chemical grouping	LOD (% < LOD)	SDs (operative/population)	Operative cohort (n = 473)	Population cohort (n = 127)	Operative cohort (n = 473)	Population cohort (n = 127)
Fat (ng/g fat)												
OCPs												
γ-HCH		0.060 (12)		0.27/—		1.35 (1.03, 1.77)		—		1.27 (1.01, 1.59)		—
PBDEs												
PBDE-47		0.100 (0)		1.034/—		0.68 (0.54, 0.86)		—		0.70 (0.55, 0.90)		—
PBDE-183		1.200 (68)		0.852/—		1.30 (1.04, 1.62)		—		1.21 (0.96, 1.52)		—
ΣPBDEs				1.055/—		0.70 (0.56, 0.88)		—		0.69 (0.54, 0.88)		—
PCBs												
PCB-28		0.065 (37)		0.519/—		1.30 (1.04, 1.62)		—		1.16 (0.92, 1.47)		—
PCB-74		0.030 (7)		0.691/—		0.77 (0.61, 0.96)		—		0.72 (0.55, 0.93)		—
PCB-151		0.030 (30)		0.099/—		1.31 (1.03, 1.67)		—		1.25 (0.99, 1.56)		—
PCB-156		0.030 (20)		0.665/—		0.78 (0.62, 0.98)		—		0.74 (0.57, 0.96)		—
PCB-201		0.030 (16)		0.170/—		1.28 (1.03, 1.60)		—		1.20 (0.94, 1.53)		—
Serum (ng/g serum)												
Cotinine		0.010 (38)		1.755/1.432		0.80 (0.66, 0.98)		0.34 (0.04, 3.12)		0.85 (0.69, 1.05)		0.34 (0.04, 3.06)
OCPs												
β-HCH		0.010 (64)		0.044/0.016		0.76 (0.54, 1.07)		1.52 (1.01, 2.29)		0.77 (0.54, 1.14)		1.72 (1.09, 2.72)
PBDEs												
ΣPBDEs				0.208/0.209		0.87 (0.71, 1.06)		1.64 (1.08, 2.50)		0.90 (0.72, 1.11)		1.58 (0.97, 2.57)
PCBs												
PCB-206		0.003 (68)		0.007/0.008		0.79 (0.65, 0.95)		1.10 (0.66, 1.83)		0.79 (0.65, 0.97)		1.19 (0.72, 1.95)
—, not applicable (no fat available for population cohort). A total of 22 women from the operative cohort were exclucded because their surgeries were canceled, and 4 women from the population cohort were excluded because their MRIs were not readable. Concentrations were log(X + 1)-transformed, rescaled by their SDs for analysis, and adjusted for age (years), BMI (continuous), breast-feeding (categorical, conditional on parity), serum cotinine (continuous), and serum lipids (continuous, milligrams per deciliter). SDs were derived for each cohort based on the log-transformed concentrations.												

Table 3 presents the logistic regression results for each chemical observed to be significantly associated with the odds of an endometriosis diagnosis in each cohort and by biological medium. Results for all chemicals not achieving significance are provided in Supplemental Material, Table 4 (http://dx.doi.org/10.1289/ehp.1104432). Several noteworthy patterns emerged, including the absence of consistent chemical effects across biological media or cohorts and a modest attenuation in the magnitude of point estimates after adjustment, although with no change in the direction of the adjusted ORs (AORs). In the operative cohort, where chemicals could be measured in fat, γ-HCH was positively associated with endometriosis (per 1-SD increase: AOR = 1.27; 95% CI: 1.01, 1.59). Of note are the three compounds associated with reduced odds of diagnosis: PBDE-47 (AOR = 0.70; 95% CI: 0.55, 0.90), PCB-74 (AOR = 0.72; 95% CI: 0.55, 0.93), and PCB-156 (AOR = 0.74; 95% CI: 0.57, 0.96). Serum β-HCH was the only POP that showed a statistically signficant association with endometriosis in the population cohort (per 1-SD increase: AOR = 1.72; 95% CI: 1.09, 2.72).

We conducted a number of sensitivity analyses to assess the robustness of our primary findings, given different modeling assumptions (data not shown). Given the uncertain role of breast-feeding and parity in the etiologic pathway, we removed it from the model and observed similar associations for fat γ-HCH (AOR = 1.26; 95% CI: 1.00, 1.58) and serum β-HCH (AOR = 1.70; 95% CI: 1.08, 2.66) in the operative and population cohorts, respectively. A reversal in the direction of the AOR was observed for fat γ-HCH when restricting the endometriosis to include visualization and histology (AOR = 0.86; 95% CI: 0.57, 1.28) and to stages 2 and 4 (AOR = 0.86; 95% CI: 0.47, 1.55). The AOR remained elevated for fat γ-HCH (AOR = 1.31; 95% CI: 0.96, 1.79) when restricting the comparison women to those with a postoperative diagnosis of a normal pelvis. We also noted an association with two other POPs in this analysis using fat concentrations: *a*) PBDE-183 (AOR = 1.55; 95% CI: 1.06, 2.26) and *b*) PCB-151 (AOR = 3.23; 95% CI: 1.43, 7.28).

## Discussion

We observed two previously unreported POPs (γ-HCH and β-HCH) to be associated with an increased odds of an endometriosis diagnosis in the operative and population cohorts, respectively. Our findings for HCH isomers were robust to adjustment, although with some reduction in magnitude. All remaining POPs varied by cohort and biological medium. Also of note is the observation that three POPs (i.e., PBDE-47, PCB-74, and PCB-156) measured in fat were inversely associated with the odds of an endometriosis diagnosis in the operative cohort.

The novel use of a matched cohort design allowed us to assess the consistency of findings by study cohort, diagnostic method, and biological medium. Our inconsistent findings across study cohorts and biological media suggest the importance of such factors when attempting to weigh available evidence. Because women in the population cohort did not undergo laparoscopies, we cannot assess chemical profiles of fat across cohorts. We did attempt to compare our omental fat concentrations with the sole previous study that quantified PCBs in serum and fat and noted lower concentrations for our cohort (Louis et al. 2005). This may reflect more nulligravid women coupled with a limited (*n* = 15) number of omental fat samples analyzed in the earlier study relative to ours, along with different laboratory analytic methods. Our findings do corroborate higher concentrations in fat relative to lipid-adjusted serum concentrations consistent with their lipophilicity (Allam and Lucena 2001; Johnson-Restrepo et al. 2005; Whitcomb et al. 2005). Fat concentrations reflect steady-state concentrations that integrate lipophilic chemicals accumulated over time. They reflect body burdens that are less affected by factors that may affect serum concentrations. Use of proxy biospecimens for lipophilic chemicals may mask or minimize health effects, as suggested by the lack of consistent findings across biological media irrespective of cohort, and may account for equivocal findings published to date.

We assessed the impact of diagnostic method for endometriosis in relation to the inconsistency of study findings across cohorts. MRI-diagnosed endometriosis may have limited sensitivity and specificity relative to visualization depending upon the presence of classical or atypical lesions and disease severity (Stratton et al. 2003). Therefore, we conducted sensitivity analyses to restrict endometriosis to stages 3 and 4 and observed no association with β-HCH in the operative cohort (AOR = 0.68; 95% CI: 0.28, 1.66). Also of note was the change in direction for γ-HCH (AOR = 0.86; 95% CI: 0.57, 1.28) when diagnosis was restricted to histologic and visualized disease. These findings suggest that γ-HCH may be associated with milder rather than more severe disease, or they may reflect the fragility of models given the reduction in power and bidirectional errors in clinical staging of endometriosis. Further study of HCH is warranted, given the relatively consistent findings for its positive association with endometriosis across cohorts, although with different isomers emerging for each cohort. Such differences may reflect the toxicologic properties (γ-HCH is more toxic than β-HCH) or bioaccumulation potential (β-HCH is more bioaccumulative than γ-HCH) of HCH isomers. The findings await future corroboration.

The emergence of two other POPs—PBDE-183 and PCB-151—when restricting the referent group to women with a postoperative report of a normal pelvis as a part of our sensitivity analyses is intriguing. This finding underscores the importance of ensuring that the comparison group undergoes surgical visualization to identify women with no endometriosis or other gynecologic pathology. This finding may suggest a possible shared etiology for endometriosis and other gynecologic disorders for some POPs, although a more complete understanding is not possible without purposefully designed research aimed at addressing this question.

We present novel findings of an association between lipophilic HCH isomers and endometriosis. HCH production and sale ceased in the United States in 2007, with earlier restrictions for agriculture use (Agency for Toxic Substances and Disease Registry 2005). γ-HCH has a shorter half-life than does β-HCH (~2 weeks and 7 years, respectively) resulting in human exposure, which may be declining except for select subpopulations (Becker et al. 2002; Centers for Disease Control and Prevention 2009; Stehr-Green 1989). Further interpretation of our findings in the context of past literature is challenging, because no two studies are directly comparable. Pregnancy and breast-feeding history affects internal dose of POPs and may result in concentration differences for women with and without visualized endometriosis. This is particularly true if the former group has fewer pregnancies and breast-feeding intervals relative to unaffected women. Because some POPs were associated with reduced fecundity (Harley et al. 2010; Meeker et al. 2011), we included a conditional breast-feeding variable in our models to adjust for reproductive histories and observed little change in the estimates. Also, serum PCB concentrations are reported to decline throughout pregnancy (Glynn et al. 2011), particularly when modeled as a function of women’s baseline exposures (Bloom et al. 2007).

We were able to locate reports of some studies that focused on OCPs but not PBDEs. Lebel et al. (1998) reported higher geometric mean concentrations for six of nine measured OCPs, including β-HCH, for women with endometriosis (*n* = 86) compared with women without endometriosis (*n* = 70), although none of the differences achieved significance, possibly a function of overmatching the comparison women on surgical indication. Trabert et al. (2010) reported comparable median serum DDE concentrations by endometriosis status consistent with our observation. Lastly, Cooney et al. (2010) reported that women in the third versus first tertile of serum HCB were significantly more likely to have surgically visualized endometriosis.

The exact mechanisms by which POPs may influence the development of endometriosis remain unknown, although several pathways have been suggested, such as potent modulation of immune and endocrine function (Rier and Foster 2002). Human endometrium is a known site for estrogen, and many POPs or their metabolites have been detected there (Schaefer et al. 2000). POPs may exert effects on estrogen or other hormonal production, or induce inflammation and the chronic stimulation of proinflammatory cytokines. Both PCBs and DDE have been associated with immunologic changes, such as the down-regulation of natural killer cells or interleukin-1β and interleukin-12 (Quaranta et al. 2006).

Our study has important limitations, including a relatively limited population cohort size, of which 11% of women were found to have endometriosis, and possible selection bias arising from the use of telephone directories for defining the population cohort recruited from California. The extent to which such directories represent the female populations for the referent population is unknown. The lack of major differences between cohorts may simply reflect who participates in research irrespective of sampling framework rather than selection factors per se. Other study limitations include the lack of quantified dioxin exposure, given its suggestive association with endometriosis (Eskenazi et al. 2002), and our inability to establish the timing and temporal ordering of fat and serum concentrations relative to development of disease, including a possible *in utero* origin (Buck Louis et al. 2010; Signorile et al. 2010). We are unaware of any human data specifying the interval between exposure and disease onset, although it was estimated to be 7–10 years in rhesus monkeys (Rier et al. 1993). The dynamic nature of endometriosis, often characterized by periods of disease progression and regression, further challenges our ability to delineate initiating events or to diagnosis truly incident disease (D’Hooghe et al. 1992; Redwine 1987).

## Conclusions

Two new POPs from the same chemical class, γ-HCH and β-HCH, were associated with increased odds of an endometriosis diagnosis in each the operative and population cohorts, respectively. The findings were largely robust to model specification and choice of referent population for the operative cohort.

## Supplemental Material

(193 KB) PDF
