# Supplementary material for: Persistent Lipophilic Environmental Chemicals and Endometriosis: The ENDO Study
Source: Environ Health Perspect. 2012 Mar 14;120(6):811–6. doi: 10.1289/ehp.1104432 (PMC3385438; doi:10.1289/ehp.1104432)
Supplement: (193 KB) PDF [file ehp.1104432.s001.pdf]

Supplemental Material

Persistent Lipophilic Environmental Chemicals and Endometriosis: The ENDO Study

Germaine M. Buck Louis, Zhen Chen, C. Matthew Peterson, Mary L. Hediger, Mary S. Croughan, Rajeshwari Sundaram, Joseph B. Stanford, Michael W. Varner, Victor Y. Fujimoto, Linda C. Giudice, Ann Trumble, Patrick J. Parsons, and Kurunthachalam Kannan

Table of Contents

Supplemental Material, Table 1. Median comparisons of persistent organochlorine pollutant concentrations (wet weight) in omentum fat by endometriosis status, The ENDO Study, 2007-2009 (n=339).

Supplemental Material, Table 2. Median comparisons of persistent organochlorine pollutant concentrations (wet weight) in serum by endometriosis status and cohort, The ENDO Study, 2007-2009 (n=600).

Supplemental Material, Table 3. Comparison of lipid adjusted chemical distributions by biologic media, cohort and endometriosis status, The ENDO Study (n=600).

Supplemental Material, Table 4. Odds ratios of an endometriosis diagnosis for chemicals not achieving significance by biologic media and cohort, The ENDO Study (n=600).

Supplemental Material, Table 1. Median comparisons of persistent organochlorine pollutant concentrations (wet weight) in omentum fat by endometriosis status in the operative cohort, The ENDO Study, 2007-2009 (n=339).

Persistent Organochlorine Pollutants (ng/g)	% <LOD	Operative Cohort (n=339)	
		Endometriosis Median (25, 75th)	No Endometriosis Median (25, 75th)
**OCPs:**			
HCB	0	5.1057 (3.3446, 9.1194)	5.1661 (3.3328, 8.6178)
γ-HCH**	13	0.1991 (0.0934, 0.3460)	0.1200 (0.0774, 0.2101)
β-HCH**	13	0.1991 (0.0934, 0.3740)	0.1200 (0.0774, 0.2160)
Oxychlordane	0	4.7029 (2.8175, 7.3500)	4.7381 (2.7251, 7.5163)
Trans-nonachlor*	2	4.4488 (2.6700, 8.7157)	5.4542 (2.9800, 8.8610)
p,p'-DDT	7	0.4400 (0.1401, 2.8400)	1.1765 (0.1300, 3.0544)
o,p'-DDT	7	0.1105 (0.0600, 0.2234)	0.0827 (0.0500, 0.1992)
p,p'-DDE	0	79.112 (46.478, 151.85)	90.886 (59.366, 165.54)
Trans-chlordane	33	0.1019 (0.0516, 0.1838)	0.0825 (0.0446, 0.1640)
Cis-chlordane	12	0.1713 (0.1089, 0.3322)	0.1928 (0.1032, 0.3233)
Cis-nonachlor	7	0.2418 (0.1100, 0.6294)	0.2900 (0.1152, 0.6587)
**PBDEs:**			
47**	0	13.467 (7.6852, 30.703)	21.589 (11.313, 51.093)
99	24	2.0484 (0.6574, 4.5980)	2.7937 (0.1882, 8.6223)
100**	17	2.7684 (0.4744, 6.9900)	4.6170 (0.8328, 7.7368)
153*	9	5.2002 (1.8708, 13.929)	7.6200 (2.6532, 18.504)
154	59	0.1787 (0.0689, 0.9565)	0.1294 (0.0045, 0.8344)
183	68	0.2317 (0.0018, 2.5900)	0.1118 (0.0007, 1.9000)
209	8	2.2540 (0.8924, 5.3566)	1.8277 (0.7248, 4.5259)
**PCBs:**			
18	47	0.0735 (0.0314, 0.1888)	0.0700 (0.0275, 0.2941)
28	37	0.1111 (0.0400, 1.3627)	0.1038 (0.0300, 0.7416)
44	33	0.0610 (0.0280, 0.1307)	0.0500 (0.0221, 0.1100)

Persistent Organochlorine Pollutants (ng/g)	& <LOD	Endometriosis Median (25, 75th)	No Endometriosis Median (25, 75th)
49	37	0.0500 (0.0258, 0.0980)	0.0387 (0.0218, 0.0769)
52	37	0.0490 (0.0256, 0.0900)	0.0354 (0.0218, 0.0745)
66	29	0.0514 (0.0294, 0.1014)	0.0500 (0.0243, 0.0919)
74	7	1.4303 (0.2339, 2.8966)	1.8628 (0.7479, 3.8220)
87*	32	0.0500 (0.0294, 0.1089)	0.0417 (0.0212, 0.0661)
99	01	0.9919 (0.6939, 1.7247)	1.0524 (0.6013, 2.0028)
101	9	0.1841 (0.0817, 0.3692)	0.1657 (0.0717, 0.3379)
118	1	1.4702 (0.9526, 2.3982)	1.4995 (0.9453, 2.8569)
128	26	0.0814 (0.0300, 0.1569)	0.0717 (0.0274, 0.1252)
138	0	4.2031 (2.5871, 6.8013)	4.1095 (2.1930, 7.5507)
146	4	0.5705 (0.2558, 1.0098)	0.5537 (0.2179, 1.0863)
149	27	0.0504 (0.0294, 0.1180)	0.0481 (0.0274, 0.0733)
151*	30	0.0500 (0.0300, 0.1100)	0.0436 (0.0245, 0.0635)
153	0	5.8892 (3.7173, 8.9910)	5.4043 (2.8416, 10.061)
156	20	0.3150 (0.0490, 1.7238)	0.7038 (0.0413, 2.0113)
157	23	0.0621 (0.0306, 0.1548)	0.0555 (0.0300, 0.1176)
167	28	0.0700 (0.0300, 0.1900)	0.0516 (0.0243, 0.1433)
170	1	1.5898 (0.7768, 3.3149)	1.4779 (0.7415, 2.6957)
172**	4	0.2458 (0.1147, 0.4143)	0.1830 (0.0897, 0.4100)
177	6	0.2210 (0.1083, 0.3922)	0.2066 (0.1023, 0.5122)
178	5	0.3248 (0.1337, 0.6614)	0.2991 (0.1048, 0.6368)
180	0	4.0214 (2.4252, 7.0617)	3.8376 (2.0085, 6.8655)
183	2	0.5684 (0.2505, 1.0530)	0.5040 (0.2426, 0.9186)
187*	0	1.5361 (0.9186, 2.4793)	1.3239 (0.7178, 2.3174)
189	15	0.0884 (0.0400, 0.1431)	0.0749 (0.0423, 0.1120)
194	10	0.5262 (0.0626, 1.3197)	0.5447 (0.0507, 1.2136)
195	10	0.1612 (0.0507, 0.4388)	0.1247 (0.0500, 0.4252)
196	2	0.7459 (0.3361, 1.2957)	0.7724 (0.4281, 1.4888)
201**	16	0.1002 (0.0478, 0.2451)	0.0600 (0.0400, 0.1867)
206	11	0.4016 (0.1165, 0.7843)	0.4761 (0.1427, 1.1765)

Persistent Organochlorine Pollutants (ng/g)	& <LOD	Endometriosis Median (25, 75th)	No Endometriosis Median (25, 75th)
209	17	0.2239 (0.0897, 0.4902)	0.2256 (0.0831, 0.4933)

*p<0.05; **p<0.01

Supplemental Material, Table 2. Median comparisons of persistent organochlorine pollutant concentrations (wet weight) in serum by endometriosis status and cohort, The ENDO Study, 2007-2009 (n=600).

Persistent Organochlorine Pollutants (ng/g)	% < LOD	Operative Cohort (n=473)		Population Cohort (n=127)	
		Endometriosis Median (25, 75th)	No Endometriosis Median (25, 75th)	Endometriosis Median (25, 75th)	No Endometriosis Median (25, 75th)
**OCPs:**					
HCB	33	0.0173 (0.0067, 0.0322)	0.0156 (0.0056, 0.0356)	0.0200 (0.0074, 0.0278)	0.0111 (0.0043, 0.0277)
γ-HCH	68	0.0057 (0.0018, 0.0156)	0.0063 (0.0024, 0.0156)	0.0069 (0.0060, 0.0156)	0.0061 (0.0014, 0.0122)
β-HCH	64	0.0063 (0.0020, 0.0134)	0.0063 (0.0029, 0.0156)	0.0066 (0.0044, 0.0098)	0.0063 (0.0027, 0.0126)
Oxychlordane	95	0.0030 (0.0008, 0.0156)	0.0036 (0.0011, 0.0156)	0.0112 (0.0021, 0.0313)	0.0057 (0.0021, 0.0163)
Trans-nonachlor	84	0.0023 (0.0006, 0.0080)	0.0029 (0.0007, 0.0076)	0.0042 (0.0025, 0.0079)	0.0039 (0.0016, 0.0075)
p,p'-DDT	89	0.0046 (0.0017, 0.0106)	0.0051 (0.0016, 0.0156)	0.0043 (0.0018, 0.0063)	0.0061 (0.0016, 0.0094)
o,p'-DDT	81	0.0029 (0.0009, 0.0112)	0.0029 (0.0009, 0.0077)	0.0031 (0.0005, 0.0070)	0.0038 (0.0009, 0.0063)
p,p'-DDE	1	0.0901 (0.0546, 0.1795)	0.1214 (0.0581, 0.2483)*	0.0962 (0.0479, 0.1918)	0.1083 (0.0639, 0.2111)
Trans-chlordane	75	0.0033 (0.0006, 0.0098)	0.0031 (0.0007, 0.0103)	0.0036 (0.0003, 0.0085)	0.0028 (0.0008, 0.0063)
Cis-chlordane	87	0.0027 (0.0003, 0.0095)	0.0021 (0.0003, 0.0073)	0.0025 (-.0002, 0.0063)	0.0023 (0.0007, 0.0063)
Cis-nonachlor	99	0.0025 (0.0007, 0.0063)	0.0027 (0.0006, 0.0063)	0.0030 (0.0017, 0.0063)	0.0028 (0.0008, 0.0063)
**PBDEs:**					
47	6	0.0379 (0.0217, 0.0698)	0.0450 (0.0216, 0.0879)*	0.0460 (0.0250, 0.0820)	0.0437 (0.0256, 0.0818)
99	9	0.0193 (0.0109, 0.0365)	0.0214 (0.0112, 0.0368)	0.0272 (0.0186, 0.0328)	0.0249 (0.0128, 0.0373)
100	20	0.0098 (0.0058, 0.0173)	0.0108 (0.0059, 0.0201)	0.0142 (0.0101, 0.0256)	0.0100 (0.0063, 0.0162)
153	5	0.0217 (0.0121, 0.0328)	0.0216 (0.0113, 0.0347)	0.0311 (0.0183, 0.0486)	0.0185 (0.0127, 0.0306)
154	36	0.0078 (0.0034, 0.0139)	0.0073 (0.0032, 0.0134)	0.0090 (0.0042, 0.0148)	0.0071 (0.0035, 0.0140)
183	95	0.0031 (0.0031, 0.0031)	0.0031 (0.0031, 0.0031)	0.0031 (0.0031, 0.0031)	0.0031 (0.0031, 0.0031)
209	6	0.0410 (0.0229, 0.1119)	0.0408 (0.0194, 0.1234)	0.0452 (0.0283, 0.4926)	0.0392 (0.0192, 0.1813)
**PCBs:**					
18	56	-0.0035 (-.0211, 0.0150)	-0.0001 (-.0209, 0.0226)	0.0007 (-.0031, 0.0264)	0.0049 (-.0094, 0.0259)
28	40	0.0058 (-.0087, 0.0280)	0.0062 (-.0040, 0.0265)	0.0233 (0.0020, 0.0433)	0.0119 (0.0020, 0.0325)
44	42	0.0050 (-.0037, 0.0237)	0.0058 (-.0018, 0.0299)	0.0113 (0.0023, 0.0287)	0.0109 (0.0006, 0.0362)
49	44	0.0041 (-.0051, 0.0162)	0.0046 (-.0022, 0.0177)	0.0079 (0.0013, 0.0169)	0.0069 (-.0008, 0.0188)
52	42	0.0083 (-.0083, 0.0545)	0.0074 (-.0057, 0.0553)	0.0078 (-.0006, 0.0540)	0.0128 (-.0035, 0.0424)
52	42	0.0083 (-.0083, 0.0545)	0.0074 (-.0057, 0.0553)	0.0078 (-.0006, 0.0540)	0.0128 (-.0035, 0.0424)

Persistent Organochlorine Pollutants (ng/g)	% <LOD	Operative Cohort (n=473)		Population Cohort (n=127)	
		Endometriosis Median (25, 75th)	No Endometriosis Median (25, 75th)	Endometriosis Median (25, 75th)	No Endometriosis Median (25, 75th)
66	43	0.0038 (-.0068, 0.0434)	0.0081 (-.0062, 0.0576)	0.0109 (0.0005, 0.0779)	0.0167 (-.0001, 0.1173)
74	51	0.0026 (-.0069, 0.0202)	0.0025 (-.0054, 0.0158)	0.0081 (-.0009, 0.0256)	0.0058 (-.0011, 0.0249)
87	51	0.0026 (-.0026, 0.0136)	0.0028 (-.0012, 0.0152)	0.0042 (-.0003, 0.0180)	0.0028 (-.0019, 0.0094)
99	46	0.0035 (-.0040, 0.0157)	0.0043 (-.0018, 0.0145)	0.0042 (0.0006, 0.0140)	0.0053 (-.0005, 0.0123)
101	45	0.0050 (-.0080, 0.0423)	0.0066 (-.0069, 0.0479)	0.0047 (-.0007, 0.0583)	0.0086 (-.0017, 0.0301)
118	48	0.0014 (-.0083, 0.0312)	0.0042 (-.0077, 0.0348)	0.0054 (-.0025, 0.0465)	0.0074 (-.0036, 0.0482)
128	72	-0.0004 (-.0026, 0.0032)	0.0004 (-.0015, 0.0038)	-0.0003 (-.0015, 0.0049)	0.0006 (-.0017, 0.0050)
138	24	0.0143 (0.0034, 0.0448)	0.0172 (0.0023, 0.0471)	0.0132 (0.0066, 0.0504)	0.0178 (0.0069, 0.0430)
146	62	0.0017 (-.0010, 0.0070)	0.0016 (-.0009, 0.0065)	-0.0002 (-.0019, 0.0061)	0.0018 (-.0011, 0.0046)
149	58	0.0016 (-.0055, 0.0180)	0.0015 (-.0040, 0.0192)	0.0022 (-.0050, 0.0096)	-0.0002 (-.0063, 0.0052)
151	64	0.0002 (-.0019, 0.0092)	0.0012 (-.0019, 0.0081)	-0.0006 (-.0019, 0.0020)	-0.0005 (-.0019, 0.0050)
153	31	0.0098 (-.0013, 0.0350)	0.0136 (0.0009, 0.0359)	0.0108 (0.0038, 0.0363)	0.0109 (0.0020, 0.0296)
156	100	0.0000 (0.0000, 0.0016)	0.0000 (0.0000, 0.0016)	0.0000 (0.0000, 0.0016)	0.0000 (0.0000, 0.0016)
157	100	0.0000 (0.0000, 0.0016)	0.0000 (0.0000, 0.0016)	0.0000 (0.0000, 0.0016)	0.0000 (0.0000, 0.0016)
167	99	0.0000 (0.0000, 0.0016)	0.0000 (0.0000, 0.0016)	0.0000 (0.0000, 0.0016)	0.0000 (0.0000, 0.0016)
170	52	0.0027 (0.0000, 0.0065)	0.0036 (0.0000, 0.0087)	0.0032 (-.0006, 0.0073)	0.0024 (0.0000, 0.0079)
172	49	0.0030 (-.0036, 0.0109)	0.0038 (-.0026, 0.0104)	0.0021 (-.0010, 0.0131)	0.0045 (-.0010, 0.0134)
177	86	0.0016 (0.0000, 0.0016)	0.0008 (0.0000, 0.0016)	0.0000 (0.0000, 0.0016)	0.0000 (0.0000, 0.0016)
178	91	0.0016 (0.0000, 0.0016)	0.0016 (0.0000, 0.0016)	0.0000 (0.0000, 0.0016)	0.0000 (0.0000, 0.0016)
180	51	0.0024 (-.0119, 0.0138)	0.0039 (-.0107, 0.0162)	-0.0002 (-.0039, 0.0109)	0.0026 (-.0133, 0.0137)
183	79	0.0016 (0.0000, 0.0019)	0.0016 (0.0000, 0.0031)	0.0016 (0.0000, 0.0016)	0.0000 (0.0000, 0.0016)
187	48	0.0037 (-.0020, 0.0093)	0.0040 (0.0000, 0.0094)	0.0034 (0.0000, 0.0055)	0.0022 (0.0000, 0.0068)
189	100	0.0000 (0.0000, 0.0016)	0.0000 (0.0000, 0.0016)	0.0000 (0.0000, 0.0016)	0.0000 (0.0000, 0.0016)
194	97	0.0000 (0.0000, 0.0016)	0.0000 (0.0000, 0.0016)	0.0000 (0.0000, 0.0016)	0.0000 (0.0000, 0.0016)
195	84	0.0012 (0.0000, 0.0021)	0.0000 (0.0000, 0.0017)	0.0000 (0.0000, 0.0016)	0.0000 (0.0000, 0.0016)
196	84	0.0016 (0.0000, 0.0021)	0.0015 (0.0000, 0.0023)	0.0000 (0.0000, 0.0016)	0.0000 (0.0000, 0.0016)
201	90	0.0000 (0.0000, 0.0016)	0.0000 (0.0000, 0.0016)	0.0000 (0.0000, 0.0016)	0.0000 (0.0000, 0.0016)
206	68	0.0012 (-.0007, 0.0038)	0.0016 (-.0005, 0.0047)	0.0027 (-.0004, 0.0048)	0.0011 (-.0005, 0.0032)
209	80	-0.0005 (-.0020, 0.0026)	0.0001 (-.0017, 0.0027)	0.0012 (-.0013, 0.0035)	-0.0003 (-.0019, 0.0015)

NOTE: Serum concentrations significant only for operative cohort. Negative concentrations stemmed from recovery-adjusted blanks.

*p<0.05, operative cohort only

Supplemental Material, Table 3. Comparison of lipid adjusted chemical distributions by biologic media, cohort and endometriosis status, The ENDO Study (n=600).

Biologic Media and Chemical Grouping (tertiles)	Operative Cohort (n=473)		Population Cohort (n=127)	
	Endometriosis	None	Endometriosis	None
**Lipid Adjusted Omentam Fat (n=340)**				
Σ OCPs (ng/g lipids):				
1st, 0.17-9.47	51 (35)	62 (32)	--	--
2nd, 9.48-17.34	49 (34)	63 (33)	--	--
3rd, 17.35-103.85	46 (32)	66 (35)	--	--
Geometric mean (95% CI)	11.35 (9.62, 13.39)	12.14 (10.71, 13.77)	--	--
Σ PBDEs (ng/g lipids):				
1st, 2.37-33.14	62 (42)**	52 (27)	--	--
2nd, 33.15-87.69	52 (35)	60 (31)	--	--
3rd, 87.70-3728.19	33 (22)	81 (42)	--	--
Geometric mean (95% CI)	45.30 (38.60, 53.17)**	66.00 (56.32, 77.34)	--	--
Σ PCBs (ng/g lipids):				
1st, 4.36-27.99	44 (30)	70 (36)	--	--
2nd, 28.00-54.46	57 (39)	55 (28)	--	--
3rd, 54.47-2164.75	45 (31)	69 (36)	--	--
Geometric mean (95% CI)	39.51 (34.16, 45.70)	38.92 (34.28, 44.20)	--	--

**Biologic Media and Chemical Grouping (tertiles)**	**Operative Cohort (n=473)**		**Population Cohort (n=127)**	
	**Endometriosis**	**None**	**Endometriosis**	**None**
**Lipid Adjusted Serum[a] (n=585)**				
Σ OCPs (ng/g lipids)				
1st, 0.01-0.80	58 (31)*	98 (35)	3 (23)*	36 (34)
2nd, 0.81-2.80	68 (37)	81 (29)	6 (46)	41 (38)
3rd, 2.81-127.76	60 (32)	100 (36)	4 (31)	30 (28)
Geometric mean (95% CI)	1.50 (1.20, 1.89)	1.58 (1.32, 1.90)	2.15 (0.76, 6.04)	1.54 (1.18, 2.02)
Σ PBDEs (ng/g lipids):				
1st, 1.55-16.125	71 (38)*	86 (31)	2 (15)*	35 (33)
2nd, 16.27-29.79	57 (31)	96 (34)	4 (31)	39 (36)
3rd, 29.79-852.14	58 (31)	97 (35)	7 (54)	33 (31)
Geometric mean (95% CI)	21.78 (19.20, 24.70)	23.16 (20.64, 25.97)	44.13 (23.81, 81.79)*	24.65 (20.66, 29.41)
Σ PCBs (ng/g lipids):				
1st, 0.29-22.71	70 (38)*	89 (32)	5 (39)*	31 (29)
2nd, 22.73-89.90	58 (31)	97 (35)	1 (8)	40 (37)
3rd, 90.82-86496.67	58 (31)	93 (33)	7 (54)	36 (34)
Geometric mean (95% CI)	42.97 (33.49, 55.13)	48.77 (39.57, 60.10)	57.33 (23.74,138.45)	51.38 (39.49, 66.85)

NOTE: Excludes 22 women in the operative cohort whose surgeries were cancelled, and 4 women in the population cohort whose MRIs were unreadable. Analyte concentrations rounded to two decimal places.

P<0.05; **P<0.01 comparing women by endometriosis status within each cohort

[a]Serum concentrations lipid adjusted = (concentration analyte/total serum lipids) x 100,000.

Supplemental Material, Table 4. Odds ratios of an endometriosis diagnosis for chemicals not achieving significance by biologic media and cohort, The ENDO Study (n=600).

Biologic Media and Chemical Groupings	SDs Operative /Population	Operative cohort OR (95% CI)	Population Cohort OR (95% CI)	Operative Cohort AOR (95% CI)	Population Cohort AOR (95% CI)
**Omental Fat (ng/g)**					
**OCPs:**					
HCB	0.726	1.13 (0.91, 1.40)	--	1.21 (0.96, 1.53)	--
β-HCH	0.498	1.09 (0.87, 1.35)	--	1.08 (0.86, 1.36)	--
Oxychlordane	0.644	1.02 (0.82, 1.26)	--	1.13 (0.87, 1.48)	--
Trans-nonachlor	0.708	0.93 (0.75, 1.15)	--	0.99 (0.77, 1.29)	--
p,p'-DDT	0.828	0.84 (0.68, 1.05)	--	0.82 (0.65, 1.05)	--
o,p'-DDT	0.33	0.97 (0.78, 1.20)	--	0.92 (0.73, 1.16)	--
p,p'-DDE	1.001	0.85 (0.68, 1.06)	--	0.88 (0.69, 1.12)	--
Trans-chlordane	0.160	1.09 (0.88, 1.36)	--	1.06 (0.84, 1.32)	--
Cis-chlordane	0.223	1.00 (0.80, 1.24)	--	0.99 (0.79, 1.25)	--
Cis-nonachlor	0.326	1.08 (0.87, 1.34)	--	1.05 (0.83, 1.32)	--
**PBDEs:**					
99	1.115	0.83 (0.67, 1.04)	--	0.84 (0.66, 1.08)	--
100	1.119	0.85 (0.68, 1.05)	--	0.83 (0.66, 1.05)	--
153	1.278	0.85 (0.69, 1.06)	--	0.78 (0.62, 0.99)	--
154	0.764	0.91 (0.73, 1.15)	--	0.95 (0.74, 1.21)	--
209	0.898	1.04 (0.84, 1.28)	--	1.01 (0.80, 1.27)	--
**PCBs:**					
18	0.321	0.93 (0.75, 1.16)	--	0.91 (0.72, 1.14)	--
44	0.321	1.04 (0.84, 1.29)	--	0.97 (0.77, 1.22)	--
49	0.103	1.17 (0.94, 1.46)	--	1.15 (0.91, 1.45)	--
52	0.089	1.10 (0.89, 1.37)	--	1.09 (0.87, 1.37)	--
66	0.200	0.93 (0.74, 1.18)	--	0.97 (0.76, 1.25)	--
49	0.103	1.17 (0.94, 1.46)	--	1.15 (0.91, 1.45)	--
52	0.089	1.10 (0.89, 1.37)	--	1.09 (0.87, 1.37)	--

Biologic Media and Chemical Groupings	SDs Operative /Population	Operative cohort OR (95% CI)	Population Cohort OR (95% CI)	Operative Cohort AOR (95% CI)	Population Cohort AOR (95% CI)
**Omental Fat (ng/g)**					
**PCBs:**					
66	0.200	0.93 (0.74, 1.18)	--	0.97 (0.76, 1.25)	--
101	0.255	1.16 (0.93, 1.45)	--	1.11 (0.88, 1.40)	--
118	0.575	0.95 (0.76, 1.18)	--	0.94 (0.73, 1.20)	--
128	0.237	0.90 (0.70, 1.15)	--	0.86 (0.65, 1.13)	--
138	0.696	1.00 (0.80, 1.24)	--	1.02 (0.77, 1.35)	--
146	0.444	1.02 (0.82, 1.26)	--	0.99 (0.76, 1.29)	--
149	0.093	1.23 (0.99, 1.54)	--	1.21 (0.96, 1.51)	--
153	0.740	1.05 (0.85, 1.30)	--	1.06 (0.80, 1.42)	--
157	0.136	1.20 (0.96, 1.49)	--	1.08 (0.85, 1.37)	--
167	0.192	0.97 (0.77, 1.21)	--	1.00 (0.78, 1.27)	--
170	0.643	1.09 (0.88, 1.35)	--	1.13 (0.85, 1.50)	--
172	0.288	1.13 (0.91, 1.41)	--	1.12 (0.87, 1.43)	--
177	0.286	1.03 (0.83, 1.27)	--	1.02 (0.79, 1.31)	--
178	0.346	1.08 (0.87, 1.34)	--	1.06 (0.82, 1.38)	--
180	0.749	1.13 (0.91, 1.40)	--	1.20 (0.89, 1.61)	--
183	0.414	1.10 (0.89, 1.37)	--	1.19 (0.91, 1.55)	--
187	0.563	1.14 (0.92, 1.42)	--	1.22 (0.92, 1.62)	--
189	0.121	1.20 (0.96, 1.49)	--	1.10 (0.87, 1.39)	--
194	0.591	1.01 (0.81, 1.25)	--	1.02 (0.80, 1.30)	--
195	0.298	1.07 (0.87, 1.33)	--	1.02 (0.81, 1.29)	--
196	0.445	0.88 (0.71, 1.10)	--	0.81 (0.61, 1.07)	--
206	0.429	0.82 (0.66, 1.03)	--	0.78 (0.60, 1.00)	--
209	0.292	0.99 (0.79, 1.22)	--	0.95 (0.75, 1.20)	--

Biologic Media and Chemical Groupings	SDs Operative /Population	Operative cohort OR (95% CI)	Population Cohort OR (95% CI)	Operative Cohort AOR (95% CI)	Population Cohort AOR (95% CI)
**Serum (ng/g)**					
**OCPs:**					
HCB	0.034/0.024	0.90 (0.74, 1.10)	1.05 (0.62, 1.76)	0.92 (0.75, 1.13)	1.00 (0.56, 1.79)
γ-HCH	0.043/0.034	0.80 (0.59, 1.10)	1.25 (0.86, 1.81)	0.81 (0.56, 1.18)	1.87 (1.04, 3.36)
Oxychlordane	0.032/0.042	1.00 (0.83, 1.21)	1.11 (0.70, 1.76)	0.99 (0.82, 1.19)	1.32 (0.73, 2.39)
Trans-nonachlor	0.022/0.023	1.03 (0.86, 1.24)	1.11 (0.69, 1.78)	1.06 (0.87, 1.28)	1.22 (0.74, 2.01)
p,p'-DDT	0.054/0.054	0.85 (0.67, 1.08)	1.01 (0.59, 1.73)	0.85 (0.65, 1.11)	1.10 (0.61, 1.97)
o,p'-DDT	0.016/0.007	1.05 (0.87, 1.25)	0.89 (0.49, 1.62)	1.03 (0.85, 1.26)	0.88 (0.46, 1.66)
p,p'-DDE	0.268/0.168	0.85 (0.68, 1.06)	1.12 (0.69, 1.82)	0.85 (0.67, 1.08)	1.32 (0.70, 2.47)
Trans-chlordane	0.029/0.024	1.14 (0.94, 1.39)	1.01 (0.59, 1.74)	1.08 (0.88, 1.32)	1.01 (0.62, 1.66)
Cis-chlordane	0.022/0.026	1.13 (0.93, 1.37)	0.91 (0.43, 1.94)	1.06 (0.87, 1.30)	0.90 (0.46, 1.76)
Cis-nonachlor	0.009/0.006	1.06 (0.88, 1.27)	0.87 (0.48, 1.58)	1.02 (0.84, 1.23)	0.98 (0.51, 1.90)
**PBDEs:**					
47	0.077/0.090	0.86 (0.70, 1.05)	1.33 (0.89, 1.99)	0.89 (0.70, 1.15)	1.03 (0.55, 1.92)
99	0.047/0.035	1.03 (0.86, 1.23)	1.22 (0.79, 1.87)	1.13 (0.92, 1.37)	1.02 (0.59, 1.77)
100	0.018/0.019	0.90 (0.74, 1.10)	1.32 (0.86, 2.01)	0.95 (0.75, 1.21)	1.09 (0.60, 1.98)
153	0.031/0.032	1.01 (0.84, 1.21)	1.15 (0.72, 1.83)	1.01 (0.82, 1.25)	1.14 (0.65, 1.97)
154	0.011/0.009	1.12 (0.93, 1.35)	1.00 (0.57, 1.74)	1.13 (0.92, 1.38)	0.86 (0.46, 1.60)
183	0.001/0.000	0.95 (0.78, 1.14)	--	0.98 (0.80, 1.21)	--
209	0.449/0.505	0.96 (0.79, 1.15)	1.16 (0.73, 1.86)	0.97 (0.80, 1.18)	1.34 (0.77, 2.33)
**PCBs:**					
18	0.292/0.196	1.07 (0.89, 1.28)	0.82 (0.26, 2.62)	0.99 (0.81, 1.20)	0.77 (0.20, 3.07)
28	0.338/0.048	1.09 (0.90, 1.32)	1.07 (0.63, 1.83)	1.03 (0.84, 1.25)	1.07 (0.60, 1.91)
44	0.085/0.041	0.98 (0.81, 1.18)	0.89 (0.49, 1.62)	0.93 (0.76, 1.13)	0.94 (0.49, 1.79)

Biologic Media and Chemical Groupings	SDs Operative /Population	Operative cohort OR (95% CI)	Population Cohort OR (95% CI)	Operative Cohort AOR (95% CI)	Population Cohort AOR (95% CI)
**Serum (ng/g)**					
**PCBs:**					
49	0.075/0.024	1.02 (0.85, 1.22)	0.92 (0.50, 1.68)	0.97 (0.80, 1.18)	0.97 (0.52, 1.82)
52	0.121/0.049	0.99 (0.82, 1.19)	0.91 (0.50, 1.65)	0.92 (0.75, 1.12)	0.93 (0.49, 1.79)
66	0.146/0.116	0.96 (0.79, 1.16)	0.88 (0.48, 1.61)	0.91 (0.75, 1.12)	0.92 (0.49, 1.75)
74	0.065/0.031	1.03 (0.86, 1.24)	0.89 (0.49, 1.60)	0.97 (0.80, 1.18)	0.94 (0.49, 1.79)
87	0.041/0.024	0.93 (0.77, 1.12)	0.84 (0.39, 1.79)	0.90 (0.73, 1.10)	0.97 (0.52, 1.80)
99	0.037/0.020	1.02 (0.85, 1.23)	0.83 (0.41, 1.68)	0.96 (0.79, 1.16)	0.93 (0.46, 1.86)
101	0.098/0.046	0.91 (0.75, 1.11)	0.96 (0.54, 1.70)	0.88 (0.72, 1.08)	1.18 (0.67, 2.08)
118	0.082/0.122	1.09 (0.91, 1.30)	1.08 (0.66, 1.76)	1.01 (0.83, 1.24)	1.06 (0.66, 1.71)
128	0.021/0.029	1.03 (0.86, 1.24)	0.91 (0.48, 1.75)	1.01 (0.83, 1.22)	0.89 (0.45, 1.76)
138	0.058/0.038	1.00 (0.83, 1.20)	0.87 (0.47, 1.62)	0.98 (0.80, 1.20)	0.80 (0.40, 1.63)
146	0.010/0.007	1.07 (0.89, 1.28)	1.03 (0.60, 1.78)	0.98 (0.81, 1.19)	0.93 (0.51, 1.69)
149	0.037/0.018	1.02 (0.85, 1.22)	1.18 (0.72, 1.92)	0.97 (0.80, 1.18)	1.29 (0.80, 2.08)
151	0.011/0.010	1.00 (0.83, 1.20)	0.88 (0.46, 1.66)	0.97 (0.80, 1.18)	0.95 (0.51, 1.77)
153	0.054/0.030	0.92 (0.76, 1.11)	1.06 (0.63, 1.80)	0.87 (0.71, 1.07)	1.13 (0.68, 1.89)
156	0.001/0.001	1.18 (0.98, 1.42)	1.04 (0.60, 1.81)	1.16 (0.95, 1.41)	0.91 (0.49, 1.67)
157	0.001/0.001	1.18 (0.98, 1.42)	1.04 (0.60, 1.81)	1.16 (0.95, 1.41)	0.91 (0.49, 1.67)
167	0.001/0.001	1.12 (0.93, 1.35)	1.04 (0.60, 1.81)	1.19 (0.97, 1.47)	0.91 (0.49, 1.67)
170	0.011/0.007	0.86 (0.69, 1.07)	0.92 (0.50, 1.68)	0.79 (0.62, 1.02)	0.72 (0.32, 1.62)
172	0.014/0.011	0.98 (0.82, 1.18)	0.81 (0.45, 1.46)	0.95 (0.78, 1.16)	1.01 (0.55, 1.87)
177	0.004/0.002	0.95 (0.79, 1.15)	0.70 (0.30, 1.62)	0.92 (0.76, 1.13)	0.60 (0.24, 1.50)
178	0.002/0.001	1.07 (0.89, 1.28)	1.00 (0.58, 1.75)	1.02 (0.84, 1.24)	0.96 (0.52, 1.77)
180	0.026/0.021	0.94 (0.78, 1.13)	0.90 (0.51, 1.61)	0.91 (0.74, 1.12)	0.77 (0.40, 1.50)

Biologic Media and Chemical Groupings	SDs Operative /Population	Operative cohort OR (95% CI)	Population Cohort OR (95% CI)	Operative Cohort AOR (95% CI)	Population Cohort AOR (95% CI)
**Serum (ng/g)**					
**PCBs:**					
183	0.005/0.002	0.99 (0.82, 1.19)	0.76 (0.37, 1.58)	0.97 (0.79, 1.18)	0.75 (0.36, 1.57)
187	0.012/0.009	0.89 (0.73, 1.08)	0.91 (0.50, 1.68)	0.87 (0.71, 1.06)	1.13 (0.67, 1.93)
189	0.001/0.001	1.03 (0.86, 1.24)	1.06 (0.61, 1.84)	1.01 (0.83, 1.22)	0.92 (0.50, 1.70)
194	0.013/0.005	0.99 (0.83, 1.20)	1.25 (0.85, 1.84)	1.05 (0.87, 1.27)	0.43 (0.03, 6.71)
195	0.041/0.017	1.16 (0.89, 1.52)	1.35 (0.92, 1.97)	1.14 (0.84, 1.54)	1.31 (0.90, 1.92)
196	0.009/0.003	0.78 (0.52, 1.19)	0.93 (0.49, 1.76)	0.70 (0.42, 1.19)	0.94 (0.46, 1.92)
201	0.016/0.013	0.93 (0.76, 1.13)	1.21 (0.82, 1.80)	0.98 (0.78, 1.23)	1.29 (0.85, 1.96)
209	0.013/0.004	0.79 (0.47, 1.34)	1.22 (0.73, 2.02)	0.73 (0.41, 1.33)	1.01 (0.56, 1.81)

NOTE: Excludes 22 women from the operative cohort whose surgeries were canceled, and 4 women from the population cohort whose MRI were not readable. Chemicals were log (x+1) transformed and rescaled by their standard deviations for analysis. Standard deviations calculated from log-transformed concentrations.

Adjusted odds ratio (AOR) including age (years), BMI (continuous), breastfeeding (categorical conditional on parity), serum cotinine (continuous), and lipids (mg/dL) in serum models.

CI, 95% confidence interval; LOD, laboratory limits of detection; OR, odds ratio; SD, standard deviation

(--) denotes not applicable; no fat obtained in population cohort.
